# Are adverse childhood experiences scores associated with heroism or villainy? A quantitative observational study of Marvel and DC Cinematic Universe characters

**DOI:** 10.1371/journal.pone.0315268

**Published:** 2025-01-15

**Authors:** Julia Wigmore, Bilal Ahmed, Gabriel Joaquino, Elke Jaibeeh Barah, Zahra Upal, Teressa Boring, Marika Lee, Eron Muel, Samantha Perry, Sandra Davidson, Carla Ginn, Carla Ferreira, Twyla Ens, Jennifer Jackson

**Affiliations:** 1 Faculty of Nursing, University of Calgary, Calgary, AB, Canada; 2 Faculty of Applied Science, School of Nursing, The University of British Columbia, Vancouver, BC, Canada; University of Toronto, CANADA

## Abstract

Many superhero and villain stories include trauma, which could influence how the public perceives the impact of trauma in their own lives. Our aim was to assess whether total Adverse Childhood Experiences (ACEs) scores were associated with heroism or villainy among Marvel and DC Characters. We watched 33 films, with a total runtime of 77 hours and 5 minutes. We scored 28 characters (19 men, 8 women, and 1 gender fluid). ACEs scores were evenly distributed across heroes/villains (U = 88, z = -.465, p = .642), Marvel/DC universes (U = 95.5, z = -0116, p = .907), and gender (U = 61, z = -.979, p = .328). There was no statistically significant correlation between ACEs scores and status (r(26) = .090, p = .65), universe (r(26) = .022, p = .91), or gender (r(26) = -.188, p = .34). We found that there was no association between ACEs scores and heroism or villainy. Thus, no one is doomed to be a villain just because of early childhood experiences.

## Introduction

Adverse Childhood Experiences (ACEs) can impact health outcomes in both childhood and adulthood [1]. Childhood development is influenced by complex social, individual, and environmental factors [2]. The influence of a challenging childhood is portrayed in popular culture, including superhero films. Superheroes and villains are influential figures in early childhood development [3]; however, all ages can be influenced by this genre [4]. Children and adults look to superheroes or villains as role models for inspiration, motivation, and general lifestyle ideas. Through observational learning, children can learn new behaviours by visualising others’ behaviour, potentially integrating certain aspects of the behaviour into their lives [3]. The influence of superhero films, which are watched by millions of people worldwide, may influence the perception of ACEs.

The purpose of this paper is to identify whether higher ACEs scores [5] in popular superheroes and villains in the *Marvel Cinematic Universe (MCU)* and *Detective Comics Expanded Universe (DCEU)* were associated with heroism or villainy.

### Background

The ACEs questionnaire was developed by Felitti, Anda [5] and Anda, Felitti [6], consisting of 10 questions, targeting a variety of potentially traumatic events that occurred before the age of 18. ACEs scores are a powerful tool to help practitioners assess developmental and behavioural risk factors; however, the ACEs questionnaire cannot assess the timing of exposures or protective factors [7]. ACEs scores cannot be considered predictive of later adverse outcomes; rather, they reflect relative risk and identify the need for additional support for children and families [7].

It is well documented that portrayals of superheroes have real-world consequences for young people. Children have sustained injuries while wearing superhero costumes [8], and higher levels of lifetime superhero exposure was associated with increased risk-taking behaviour [9]. Idolizing superheroes in childhood was associated with more traditional beliefs on attractiveness and masculinity later in life [10]. Preschoolers who watched superhero programmes were associated with physical and relational aggression [11]. These studies indicate that superheroes are not only for entertainment and can impact children in real ways.

Other authors have studied the portrayal of trauma among superheroes. Sandifer [12] discussed the traumatic comic book origin stories by analysing the murder of Batman’s parents, the murder of Spider-Man’s uncle, and the destruction of Superman’s home planet. Superhero comics are a “complex network of obsession and repression organised around traumatic incidents, requiring a model that is based not on traditional narrative arcs, but on psychological structures of memory and repetition that stem from the origin trauma” [12]. Reflection on superhero narratives can help children overcome trauma, with children reengaging in post-traumatic play therapy when a superhero theme was incorporated [13]. Superhero themed interventions can enhance self-esteem among children experiencing vulnerability [14], and reading superhero-themed books improved behaviour in children with absent parents [15]. The complex depictions of superheroes’ trauma responses have the potential to influence the perceptions of children who are also experiencing trauma.

### Superhero universes

In the current study, we compared depictions of superheroes from the *MCU* and *DCEU*. Marvel was originally founded by Martin Goodman in 1939 as the comic book company Timely Comics [16]. The company changed its name to Marvel in the 1960s after hiring artists and writers Joe Simon, Jack Kirby, and Stan Lee [16]. These artists were the creators of the popular heroes known today such as Captain America and Spiderman [17]. The *MCU* was created in 2008 to maintain consistency and rebrand Marvel films into one single timeline, with its first film being Iron Man [18]. In contrast, Malcolm Wheeler-Nicholson founded *Detective Comics (DC) Inc*. in 1937 [19]. *DC* published the first Superman comic in Action Comics no. 1 in 1938, which led to the official formation of the superhero genre [19]. The *DC* series introduced Batman in 1939 and Wonder Woman in 1941. In a modern context, heroes like Batman have provided significant social commentary [20]. A significant proportion of the fanbase for these superheroes and villains in the *DCEU* and *MCU* are children who internalise the struggles, adversities, and behaviours of the characters to navigate through their own special circumstances and adversities. In the current study, we selected characters from the *MCU* and *DCEU* to ensure that the timeline and characters remained largely consistent [3].

### Research questions

Our research questions were: Are higher ACEs scores associated with villains? Are there differences between *MCU* and *DCEU* ACEs outcomes? Are there differences in ACEs scores between female and male characters?

### Hypothesis

We hypothesised that increased ACEs scores would be positively associated with characters villains and lower ACEs scores would be positively associated with heroism. We predicted no differences between the *MCU* and *DCEU*, and no difference between females and males.

## Methods

### Design

We conducted a quantitative observational study of ACEs scores on popular heroes and villains in the *MCU* and *DCEU*. We collected data by viewing selected films that revealed information about a character’s childhood and completing the ACEs questionnaire [S1 Appendix].

### Sample and participants

We chose *MCU* and *DCEU* characters who had been featured in blockbuster films in the past 20 years. We conducted a sample size calculation with the parameters of significance set at p<0.005 and a confidence interval of 80%. With a standard deviation of 2, our minimum detectable effect was seven. Thus, we included seven heroes and villains from each of the *MCU* and *DCEU* (n = 28).

As a group, we reviewed the list of *MCU* and *DCEU* films from the last 20 years, and identified which characters were depicted with information about their childhoods that we could complete the ACEs questionnaire. Some characters initially selected within the pool were excluded if we did not have any sufficient childhood information. For example, Thanos is arguably the biggest villain in recent *MCU* films, but there was insufficient information within the selected movies to complete an ACEs questionnaire, so he was excluded. We also excluded characters where most of their childhood information was available as a TV series, rather than a film (such as Scarlet Witch), to keep the viewing requirements manageable for the research team. Other characters, such as sidekicks (i.e., Robin), were also excluded due to the lack of context and childhood information given to complete the ACE questionnaire within the respective movies that were viewed.

In our study, we omitted gathering more characters from comic books in both Marvel and DC universes, due to their inconsistency in character development. Comic book storylines often feature alternative plotlines, character arcs, and multiverse outcomes. This storytelling makes comic book characters highly inconsistent and challenging to score.

### Classification as hero or villain

We defined “hero” as a character who aims to protect the public from harm, as the protagonists in their respective films. We defined “villain” as a character who aims to cause harm, or achieve their ends despite any harm caused, as the antagonists in their respective films.

Some characters were more difficult to categorize than others. Harley Quinn has been featured as a villain but became a hero after ending her relationship with the Joker, so we classified her as a hero. Loki, the God of Mischief, moved between hero and villainy repeatedly, often several times in the same film. In their self-named TV series, Loki also moves back to alternate childhoods on separate occasions. For ease of classification, we assessed Loki’s childhood as depicted in the first Thor film, and classified Loki as a villain, given that he spends more of his Marvel film character arc as a villain than a hero.

## Measure

The ACEs questionnaire is a unidimensional 10-item scale, with yes/no scoring, that identifies the relative adversity present in one’s childhood [5] (see S1 Appendix). The questions ask about experiences of neglect, abuse, and witnessing violent events, such as domestic violence. Each item/question can be answered yes (1) or no (0), with a potential score ranging between 0–10; an ACEs score of four or higher is associated with poorer health outcomes in adulthood [5].

Dube, Williamson [21] assessed the test-retest reliability of the ACEs questionnaire using responses from 608 participants across three-time points. The authors found the weighted-kappa coefficient of the total ACEs scores (range: 0–8) was 0.64 (95% CI 0.36–0.60). The validity of ACEs scores has been difficult to ascertain due to the retrospective nature of the questionnaire, with time lapses between event and reporting, variable responses due to the sensitive nature of the questions, and the link between trauma and altered memories [21]. As we completed the scoring immediately after watching the films, these concerns are less relevant in the current study. While we are not aware of previous ACEs scoring with a superhero population, the scale has been widely used in health research [22]. We also recorded the character’s status (villain or hero), universe (*MCU* and *DCEU*), and perceived gender (female or male).

### Scoring

We divided the selected films where two researchers scored each character independently. We completed the 10-item ACEs score at the end of the films. The total ACEs score was the primary outcome measure. When there was more than two points difference between the scores, a third researcher viewed the films as a tie breaker. Our scoring data are available as S2 Appendix.

### Data analysis

We used the Statistical Package for the Social Sciences (SPSS) version 29 to analyse the data. We applied descriptive statistics and the Mann-Whitney U test to determine the distribution of ACEs scores across universes, status, and gender. Due to the unknown distribution of ACEs scores, we performed the non-parametric test correlational analysis using Spearman’s rank order correlation (Spearman’s rho), to determine the correlation between ACEs scores and the demographic variables. We ensured all the assumptions of both the Mann-Whitney [23] and Spearman’s rho [24] were met. Our data are available on request.

### Ethical considerations

As these films are in the public domain, ethical approval was not required from our institution’s Research Ethics Board.

## Results

### Participants

We watched 33 films, with a total runtime of 77 hours and 5 minutes. There were eight female characters, 19 males, and one gender-fluid character (see Table 1).

**Table 1:** Demographic information.

Character	Films Included	Gender
**Marvel Villains**		
Harry Osborn	Spider-Man 3 (2007, 139 min) and The Amazing Spider-Man 2 (2014, 144 min)	Male
Hela	Thor: Ragnarock (2017, 130 min)	Female
Ivan Vanko	Iron Man 2 (2010, 124 min)	Male
Killmonger	Black Panther (2018, 134 min)	Male
Loki	Thor (2011, 115 min) and Thor: Dark World (2013, 112 min)	Gender fluid [male]
Namor	Black Panther: Wakanda Forever (2022, 161 min)	Male
Task Master	Black Widow (2021, 132 min)	Female
**Marvel Heroes**		
Black Widow	Avengers: Infinity War (2018, 149 min) and Black Widow (2021, 132 min)	Female
Hulk	Hulk (2003, 138 min) and The Incredible Hulk (2008, 112 min)	Male
Iron Man	Iron Man (2008, 126 min) and Iron Man 2 (2010, 124 min)	Male
Shang-Chi	Shang-Chi and the Legend of the 10 Rings (2021, 132 min)	Male
Shuri	Black Panther (2018, 134 min) and Black Panther: Wakanda Forever (2022, 161 min)	Female
Spiderman	Spider-Man (2002, 121 min), The Amazing Spider-Man (2012, 136 min), and Spider-Man: Homecoming (2017, 133 min)	Male
Starlord	Guardians of the Galaxy Vol. 2 (2017, 136 min)	Male
**DC Villains**		
Joker	The Dark Knight (2008, 152 min)	Male
Dr. Sivana	Shazam! (2019, 132 min)	Male
Harley Quinn	Birds of Prey (2020, 109 min) and Suicide Squad (2016, 123 min)	Female
Huntress	Birds of Prey (2020, 109 min)	Female
Maxwell Lord	Wonder Woman 1984 (2020, 151 min)	Male
Ocean Master	Aquaman (2018, 143 min)	Male
Riddler	The Batman (2022, 176 min)	Male
**DC Heroes**		
Aquaman	Aquaman (2018, 143 min)	Male
Batman	Batman Begins (2005, 140 min) and The Batman (2022, 176 min)	Male
Cat Woman	The Batman (2022, 176 min) and The Dark Knight Rises (2012, 164 min)	Female
Cyborg	Batman vs Superman: Dawn of Justice (2016, 151 min) and Zack Snyder’s Justice League (2021, 242 min)	Male
Shazam (Billy Batson)	Shazam! (2019, 132 min) and Shazam! Fury of the Gods (2023, 130 min)	Male
Superman	Superman Returns (2006, 154 min) and Man of Steel (2013, 143 min)	Male
Wonder Woman	Wonder Woman (2017, 141 min) and Wonder Woman 1984 (2020, 151 min)	Female

Loki is canonically gender fluid, but because he spends most of his time in the Thor films identifying as a man, we included him as ‘male’ for the purposes of statistical analysis. Descriptive statistics are shown in Table 2.

**Table 2:** Descriptive statistics.

						Percentiles		
	*N*	*Mean*	*Std*. *Deviation*	*Min*.	*Max*.	*25* ^*th*^	*50* ^*th*^	*75th*
**ACE**	28	3.43	1.952	0	8	2.00	3.50	5.00
**Status**	28	1.50	.509	1	2	1.00	1.50	2.00
**Universe**	28	1.50	.509	1	2	1.00	1.50	2.00
**Gender**	28	1.71	.460	1	2	1.00	1.50	2.00

### Question 1: Do scores associate with villainy or heroism?

Spearman’s rho was calculated to assess non-parametric correlation between ACEs scores and the status of the characters, their cinematic universe, and gender (Table 3). There were no statistically significant correlations between ACEs scores and status (r(26) = .090, p = .65), universe (r(26) = .022, p = .91), or gender (r(26) = -.188, p = .34). There was not enough evidence to accept our hypothesis that ACEs scores are associated with villainy or heroism.

**Table 3:** Spearman’s rho correlation.

		ACE	Gender	Universe	Status
**ACE**	Correlation Coefficient	1.000	-.188	.022	.090
	Sig. (2-tailed)	.	.337	.910	.650
	N	28	28	28	28
**Gender**	Correlation Coefficient	-.188	1.000	.000	.000
	Sig. (2-tailed)	.337	.	1.000	1.000
	N	28	28	28	28
**Universe**	Correlation Coefficient	.022	.000	1.000	.000
	Sig. (2-tailed)	.910	1.000	.	1.000
	N	28	28	28	28
**Status**	Correlation Coefficient	.090	.000	.000	1.000
	Sig. (2-tailed)	.650	1.000	1.000	.
	N	28	28	28	28

### Question 2: Are there differences in ACEs scores between groups?

**Fig 1:**
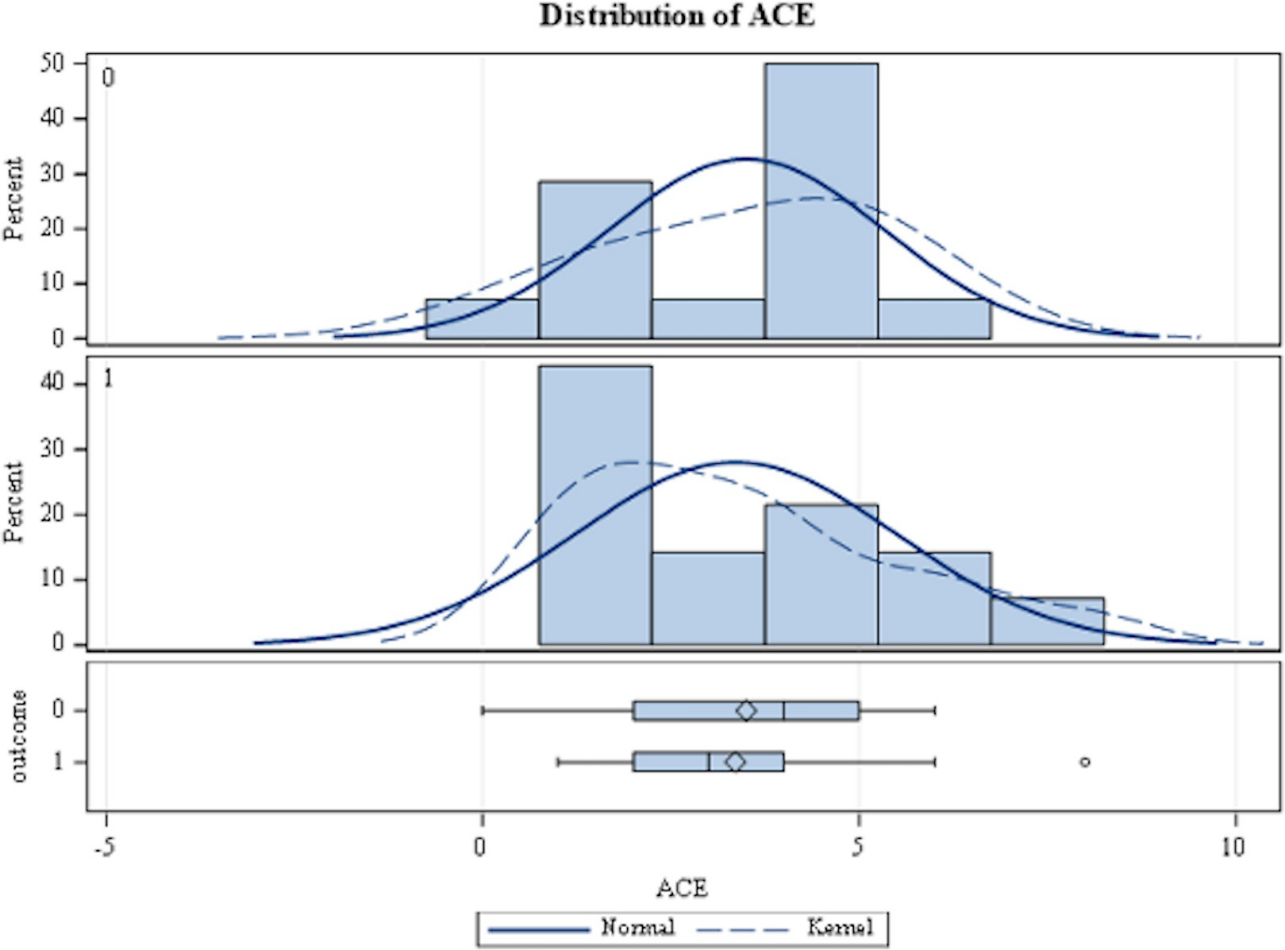
Distribution of ACEs scores across heroes and villains (exported from Statistical Analysis Software), where 0 = heroes and 1 = villains in the outcome data. The distribution of total ACE scores among heroes are negatively skewed, and total ACE scores among villains are positively skewed.

To assess the distribution of ACEs scores across status, universe, and gender, we conducted three Mann-Whitney U Tests. Although ACEs score were not normally distributed (see S3 Appendix), there were no significant differences between the groups of heroes/villains (U = 88, z = -.465, p = .642), *MCU/DCEU* (U = 95.5, z = -0116, p = .907), or males/females (U = 61, z = -.979, p = .328) at a significance level of 0.05 (See Table 4 and Fig 1). There was no significant difference in ACEs scores between heroes and villains, the *MCU* and *DCEU*, or males and females.

**Table 4:** Independent-samples Mann-Whitney U test summary.

	Status*	Universe*	Gender*
**Total N**	28	28	28
**Mann-Whitney**	88.00	95.50	61.00
**Z score**	-.465	-.116	-.979
**Asymptomatic Sig. (2-sided test)**	.642	.907	.328

*Denotes grouping variable

## Discussion

We reject our first hypothesis, as we found insufficient evidence of an association between higher ACEs score and villainy. This result is reflected in several character trajectories, such as The Joker, who had an ACEs score of six, and wreaked havoc across Gotham City. However, Black Widow has an ACEs score of eight, and she fought Thanos alongside The Avengers. Admittedly, Black Widow had a period of villainy until she became a force for good. However, Black Widow represents resilience of characters who have experienced trauma. Socio-ecologic resilience (including access to social relationships and supportive communities) can play a mitigating role in the effect of ACEs [25, 26]. Our study reinforces the longstanding message that people who experience trauma are capable of resilience [27], and that supportive environments can help people to thrive.

The ACEs questionnaire may also have been unable to detect any existing differences between heroes and villains. The ACEs questionnaire has been critiqued for its simplicity, with calls to widen the ACEs definitions of trauma to include things like natural disasters and warfare [28, 29]. A broader definition of trauma may be helpful to account for superhero behaviour among characters like Namor, who grew up during the violent colonisation of his home. There were revisions to variations of the ACEs questionnaire to be more inclusive of low-income nations [30, 31], which may support more inclusive assessments. Characters from the *MCU* and *DCEU* would benefit from a complete psychological assessment, to include screening measures beyond the ACEs questionnaire [32].

We accept our second hypothesis, as there was no statistically significant difference between ACEs scores in characters in the *MCU* and *DCEU*. Superhero films in general have been criticized for portraying characters as one-dimensional, losing the nuance present in the comic books [33]. The effort to create blockbuster juggernauts has led to pandering to cinemagoers [33, 34]. The good versus evil narratives that are central to the *MCU* and *DCEU* may reinforce homogeneity in character experiences in the films.

We accept our third hypothesis, that there is no significant difference among ACEs scores based on gender. Of the total characters studied, only two in seven were female. It could be that the limited portrayals of women in superhero films limited the representation of experiences of female characters. It is well documented that portrayals of female superheroes are hypersexualised [35], despite increasing representation of women as superheroes [36]. Such portrayals can have negative impacts on women’s self-esteem [37]. Having additional female characters, with more nuanced portrayals, in future samples may make it possible to explore gendered influences on ACEs scores.

The results of this study indicate that superheroes can be influential as role models for children. Anderson and Cavallaro [38] suggest that children tend to seek real-life figures, such as parents or teachers, as suitable role models rather than celebrities or fictional characters. This preference signifies children’s ability to critically evaluate media figures, recognizing that such characters may not embody positive traits in the real world. One child in their study noted, “when nobody’s paying attention, they do something bad,” indicating an awareness that media personalities may not be worthy of admiration. Additionally, Anderson and Cavallaro [38] discuss how superheroes influence the shaping of children’s perceptions of good and evil, often noting that superhero play involves imitating power and aggression, particularly amongst boys. They suggest that children, despite these influences, are not passive consumers of media. Even when exposed to negative characters such as villains, they can reflect critically on what they see. Clinicians could use fictional characters to illustrate to children that they can be heroes despite challenging situations [38].

Children who experience challenging or abusive upbringings, are still capable of critically selecting role models based on values and traits they see as positive, rather than blindly mimicking negative figures from their environment or media. Johnson et al. [39] supports these results, showing that children choose role models based on how they treat others, valuing traits like kindness and fairness. Therefore, children who present higher ACE scores may be inspired by examples that demonstrate that no one is doomed to become villains, as they are able to distinguish between fictional portrayals and real-world behaviours. Children present critical thinking skills that demonstrate resilience and the ability to make thoughtful choices about whom to follow, which could emphasize the utility of superheroes as role models.

There are mixed outcomes for ACEs associated with gender. In a nationally representative sample [40], ACEs scores were consistently higher for females than males; females had higher odds of ACEs scores of 4 or greater than males [41]. However, international results suggest that males experience more ACEs [42]. This evidence reinforces that while there may be gendered distinctions in childhood experiences, context is a primary factor in outcomes.

### Limitations

Our study is limited by our assessment of films, excluding TV series and other properties. The films provided a more accessible, manageable, and consistent storylines for the purposes of this study. Comic books present different and inconsistent iterations and origin stories of heroes and villains, making it difficult to apply a consistent ACEs score to a character. Scores may have been different if we considered other media sources including comic books.

No superheroes or villains were involved in this research study. If anyone could connect us with them, we would be happy to conduct a follow up study to overcome this limitation.

### Significance and next steps

Our study suggests that there are more variables to consider when exploring how early childhood experiences influence health outcomes, behaviours, and choices later in life. Although ACEs scores have been widely used in health research and practice [22], our results reinforce that these scores cannot be considered correlated with deviant or villainous behaviours; rather, they indicate a potential risk and an opportunity for additional support for children and families [7]. In future research, it may be worth exploring other factors that influence characters’ resilience and ability to rise to heroism in the face of adversity experienced in childhood.

## Conclusion

The results of this study show no statistically significant correlation between ACEs scores and villainy or heroism in either the *MCU* or *DCEU*. Thus, ACEs may not have a noteworthy impact on whether someone becomes a villain or a hero. Our findings strengthen the argument that ACEs questionnaires do not predict adverse events but instead act as a screening tool to highlight potential risks and areas for support. Depicting outcomes related to ACEs may be influential for film viewers, who see stories of both resilience and villainy resulting from traumatic experiences, and future research should explore how superhero narratives provide valuable inspiration to children and adults.

## Supporting information

S1 Checklist STROBE statement—checklist of items that should be included in reports of observational studies. (DOCX)

S1 Appendix ACES questionnaire. (DOCX)

S2 Appendix Marvel and DC ACES score spreadsheet. (XLSX)

S3 Appendix Mann Whitney U test summary and frequency tables. (DOCX)
