# Supplementary material for: Are adverse childhood experiences scores associated with heroism or villainy? A quantitative observational study of Marvel and DC Cinematic Universe characters
Source: PLoS One. 2025 Jan 15;20(1):e0315268. doi: 10.1371/journal.pone.0315268 (PMC11734917; doi:10.1371/journal.pone.0315268)
Supplement: S1 Checklist — (DOCX) [file pone.0315268.s001.docx]

STROBE Statement—checklist of items that should be included in reports of observational studies

|  | | | Item No. | Recommendation | Page  No. | | | Relevant text from manuscript |
| --- | --- | --- | --- | --- | --- | --- | --- | --- |
| **Title and abstract** | | | 1 | (*a*) Indicate the study’s design with a commonly used term in the title or the abstract | 1 | | | Are Adverse Childhood Experiences Scores Associated with Heroism or Villainy? A Quantitative Observational Study of Marvel and DC Cinematic Universe Characters |
|  |  |  |  | (*b*) Provide in the abstract an informative and balanced summary of what was done and what was found | 1 | | | see submission |
| Introduction | | | | | | | |  |
| Background/rationale | | | 2 | Explain the scientific background and rationale for the investigation being reported | 4-6 | | | Introduction and background provided |
| Objectives | | | 3 | State specific objectives, including any prespecified hypotheses | 6 | | | Our research questions were: Are higher ACEs scores associated with villains? Are there differences between MCU and DCEU ACEs outcomes? Are there differences in ACEs scores between female and male characters?  Hypothesis  We hypothesised that increased ACEs scores would be positively associated with characters villains and lower ACEs scores would be positively associated with heroism. We predicted no differences between the MCU and DCEU, and no difference between females and males. |
| Methods | | | | | | | |  |
| Study design | | | 4 | Present key elements of study design early in the paper | 7 | | | We conducted a quantitative observational study of ACEs scores on popular heroes and villains in the MCU and DCEU. We collected data by viewing selected films that revealed information about a character’s childhood and completing the ACEs questionnaire. |
| Setting | | | 5 | Describe the setting, locations, and relevant dates, including periods of recruitment, exposure, follow-up, and data collection | 7-9 | | | see submission |
| Participants | | | 6 | (*a*) *Cohort study*—Give the eligibility criteria, and the sources and methods of selection of participants. Describe methods of follow-up  *Case-control study*—Give the eligibility criteria, and the sources and methods of case ascertainment and control selection. Give the rationale for the choice of cases and controls  *Cross-sectional study*—Give the eligibility criteria, and the sources and methods of selection of participants | 7-9 | | | We chose MCU and DCEU characters who had been featured in blockbuster films in the past 20 years. We conducted a sample size calculation with the parameters of significance set at p<0.005 and a confidence interval of 80%. With a standard deviation of 2, our minimum detectable effect was seven. Thus, we included seven heroes and villains from each of the MCU and DCEU (n=28). |
|  |  |  |  | (*b*) *Cohort study*—For matched studies, give matching criteria and number of exposed and unexposed  *Case-control study*—For matched studies, give matching criteria and the number of controls per case |  | | | N/A |
| Variables | | | 7 | Clearly define all outcomes, exposures, predictors, potential confounders, and effect modifiers. Give diagnostic criteria, if applicable | 7-9 | | | see submission |
| Data sources/ measurement | | | 8* | For each variable of interest, give sources of data and details of methods of assessment (measurement). Describe comparability of assessment methods if there is more than one group | 8, appendix A | | | The ACEs questionnaire is a unidimensional 10-item scale, with yes/no scoring, that identifies the relative adversity present in one’s childhood5 (see Appendix A). |
| Bias | | | 9 | Describe any efforts to address potential sources of bias | 9 | | | When there was more than two points difference between the scores, a third researcher viewed the films as a tie breaker. |
| Study size | | | 10 | Explain how the study size was arrived at | 7 | | | We conducted a sample size calculation with the parameters of significance set at p<0.005 and a confidence interval of 80%. With a standard deviation of 2, our minimum detectable effect was seven. Thus, we included seven heroes and villains from each of the MCU and DCEU (n=28). |
| Quantitative variables | | 11 | | Explain how quantitative variables were handled in the analyses. If applicable, describe which groupings were chosen and why | 8 | | The ACEs questionnaire is a unidimensional 10-item scale, with yes/no scoring, that identifies the relative adversity present in one’s childhood (see Appendix A). We also recorded the character's status (villain or hero), universe (MCU and DCEU), and perceived gender (female or male). | |
| Statistical methods | | 12 | | (*a*) Describe all statistical methods, including those used to control for confounding | 9 | | We applied descriptive statistics and the Mann-Whitney U test to determine the distribution of ACEs scores across universes, status, and gender. Due to the unknown distribution of ACEs scores, we performed the non-parametric test correlational analysis using Spearman’s rank order correlation (Spearman’s rho), to determine the correlation between ACEs scores and the demographic variables. | |
|  |  |  |  | (*b*) Describe any methods used to examine subgroups and interactions | N/A | |  | |
|  |  |  |  | (*c*) Explain how missing data were addressed | N/A | |  | |
|  |  |  |  | (*d*) *Cohort study*—If applicable, explain how loss to follow-up was addressed  *Case-control study*—If applicable, explain how matching of cases and controls was addressed  *Cross-sectional study*—If applicable, describe analytical methods taking account of sampling strategy | N/A | |  | |
|  |  |  |  | (*e*) Describe any sensitivity analyses | N/A | |  | |
| Results | | | | | | | | |
| Participants | | 13* | | (a) Report numbers of individuals at each stage of study—eg numbers potentially eligible, examined for eligibility, confirmed eligible, included in the study, completing follow-up, and analysed | 10 | | Table 1 | |
|  |  |  |  | (b) Give reasons for non-participation at each stage | N/A | |  | |
|  |  |  |  | (c) Consider use of a flow diagram | N/A | |  | |
| Descriptive data | | 14* | | (a) Give characteristics of study participants (eg demographic, clinical, social) and information on exposures and potential confounders | 10 | | Tables 1, 2 | |
|  |  |  |  | (b) Indicate number of participants with missing data for each variable of interest | N/A | |  | |
|  |  |  |  | (c) *Cohort study*—Summarise follow-up time (eg, average and total amount) | N/A | |  | |
| Outcome data | | 15* | | *Cohort study*—Report numbers of outcome events or summary measures over time | N/A | |  | |
|  |  |  |  | *Case-control study—*Report numbers in each exposure category, or summary measures of exposure | N/A | |  | |
|  |  |  |  | *Cross-sectional study—*Report numbers of outcome events or summary measures | 11 | | Table 2 | |
| Main results | | 16 | | (*a*) Give unadjusted estimates and, if applicable, confounder-adjusted estimates and their precision (eg, 95% confidence interval). Make clear which confounders were adjusted for and why they were included | 11-13 | |  | |
|  |  |  |  | (*b*) Report category boundaries when continuous variables were categorized | N/A | |  | |
|  |  |  |  | (*c*) If relevant, consider translating estimates of relative risk into absolute risk for a meaningful time period | N/A | |  | |
| Other analyses | 17 | | Report other analyses done—eg analyses of subgroups and interactions, and sensitivity analyses | | N/A |  | | |
| Discussion | | | | | | | | |
| Key results | 18 | | Summarise key results with reference to study objectives | | 11-13 | Tables 3, 4 | | |
| Limitations | 19 | | Discuss limitations of the study, taking into account sources of potential bias or imprecision. Discuss both direction and magnitude of any potential bias | | 15 | Comic books present different and inconsistent iterations and origin stories of heroes and villains, making it difficult to apply a consistent ACEs score to a character. Scores may have been different if we considered other media sources including comic books. | | |
| Interpretation | 20 | | Give a cautious overall interpretation of results considering objectives, limitations, multiplicity of analyses, results from similar studies, and other relevant evidence | | 13-15 | see submission | | |
| Generalisability | 21 | | Discuss the generalisability (external validity) of the study results | | 13-15 | see submission | | |
| Other information | | |  | | | | | |
| Funding | 22 | | Give the source of funding and the role of the funders for the present study and, if applicable, for the original study on which the present article is based | | N/A |  | | |

*Give information separately for cases and controls in case-control studies and, if applicable, for exposed and unexposed groups in cohort and cross-sectional studies.

**Note:** An Explanation and Elaboration article discusses each checklist item and gives methodological background and published examples of transparent reporting. The STROBE checklist is best used in conjunction with this article (freely available on the Web sites of PLoS Medicine at http://www.plosmedicine.org/, Annals of Internal Medicine at http://www.annals.org/, and Epidemiology at http://www.epidem.com/). Information on the STROBE Initiative is available at www.strobe-statement.org.
