# Supplementary material for: Are adverse childhood experiences scores associated with heroism or villainy? A quantitative observational study of Marvel and DC Cinematic Universe characters
Source: PLoS One. 2025 Jan 15;20(1):e0315268. doi: 10.1371/journal.pone.0315268 (PMC11734917; doi:10.1371/journal.pone.0315268)
Supplement: S1 Appendix — (DOCX) [file pone.0315268.s002.docx]

# Appendix A: ACES Questionnaire

While you character was growing up, during their first 18 years of life:

1. Did a parent or other adult in the household often… swear at you, insult you, or humiliate you? Or act in a way that may you afraid that you might be physically hurt?
2. Did a parent or other adult in the household often…push, grab, slap, or throw something at you? Or ever hit so hard that you had marks or were injured?
3. Did and adult or person at least 5 years older than you ever… touch or fondle you or have you touch their body in a sexual way? Or try to or actually have oral, anal, or vaginal sex with you?
4. Did you often feel that… no one in your family loved you or thought you were important or special? Or your family didn’t look out for each other, feel close to each other, or support each other?
5. Did you often feel that… you didn’t have enough to eat, had to wear dirty clothes, and had no one to protect you? Or your parents were too drunk or high to take care of you or to take you to the doctor if you needed it?
6. Were your parents ever separated or divorced? Or did you experience a parental death?
7. Was one of your parents/step-guardians/guardians: often pushed, grabbed, slapped, or had something thrown at them? Or sometimes or often kicked, bitten, hit with a fist, or hit with something hard? Or ever repeatedly hit over at least a few minutes or threatened with a gun or knife?
8. Did you live with anyone who was a problem drinker or alcoholic or used street drugs or cannabis?
9. Was a household member depressed or mentally ill or did a household member attempt suicide?
10. Did a household member go to prison?
