# Supplementary material for: Are adverse childhood experiences scores associated with heroism or villainy? A quantitative observational study of Marvel and DC Cinematic Universe characters
Source: PLoS One. 2025 Jan 15;20(1):e0315268. doi: 10.1371/journal.pone.0315268 (PMC11734917; doi:10.1371/journal.pone.0315268)
Supplement: S3 Appendix — (DOCX) [file pone.0315268.s004.docx]

# Appendix B: Mann Whitney U Test Summary and Distribution Tables

## Hypothesis 1: ACE Scores are the same across heroes and villains.

| Table B1: Hypothesis Summary of ACE Distribution Across Heros/Villains | | | |
| --- | --- | --- | --- |
| **Null Hypothesis** | **Test** | **Sig.^a,b^** | **Decision** |
| The distribution of ACE scores is the same across the category of status (heroes/villains). | Independent Samples Mann-Whitney Test. | .642 | Retain the null hypothesis. |
| a. The significance level is .050. |  |  |  |
| b. Asymptotic significance is displayed. |  |  |  |
| c. Exact significance is displayed for this test. |  |  |  |


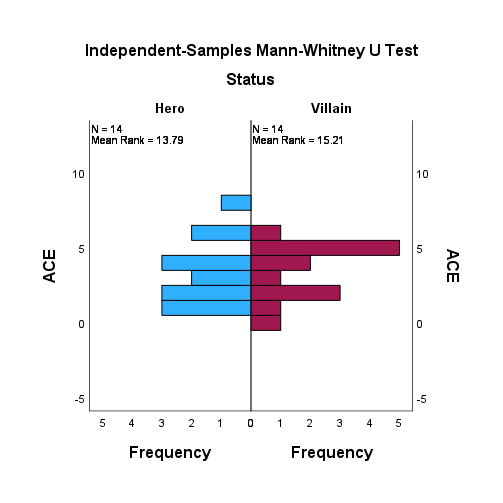


*Figure B1*. Distribution of ACE Scores across heroes and villains (exported from SPSS).

## Hypothesis 2: ACE Scores are the same across Marel and DC universes.

| Table B2: Hypothesis Summary of ACE Distribution Across Marvel/DC universes | | | |
| --- | --- | --- | --- |
| **Null Hypothesis** | **Test** | **Sig.^a,b^** | **Decision** |
| The distribution of ACE scores is the same across the universes (Marvel/DC). | Independent Samples Mann-Whitney Test. | .907 | Retain the null hypothesis. |
| a. The significance level is .050. |  |  |  |
| b. Asymptotic significance is displayed. |  |  |  |
| c. Exact significance is displayed for this test. |  |  |  |

##
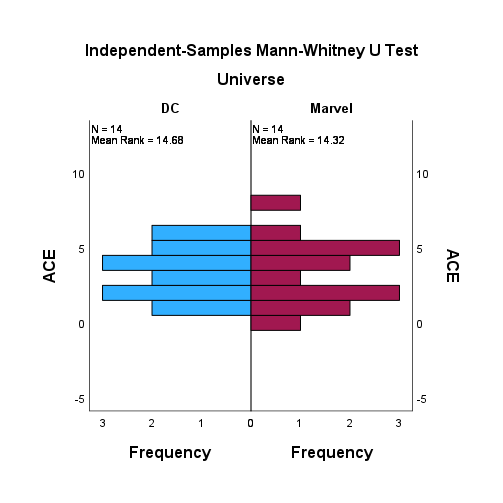


*Figure B2.* Distribution of ACE Scores across Marvel and DC universes (exported from SPSS).

## Hypothesis 3: ACE Scores are the same across gender.

| Table B3: Hypothesis Summary of ACE Distribution Across Gender | | | |
| --- | --- | --- | --- |
| **Null Hypothesis** | **Test** | **Sig.^a,b^** | **Decision** |
| The distribution of ACE scores is the same across gender. | Independent Samples Mann-Whitney Test. | .328 | Retain the null hypothesis. |
| a. The significance level is .050. |  |  |  |
| b. Asymptotic significance is displayed. |  |  |  |
| c. Exact significance is displayed for this test. |  |  |  |


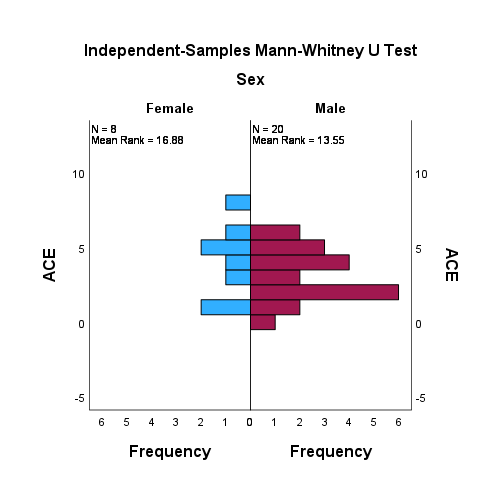


*Figure B3.* Distribution of ACE Scores across gender (exported from SPSS).
